# Parenting With a Kind Mind: Exploring Kindness as a Potentiator for Enhanced Brain Health

**DOI:** 10.3389/fpsyg.2022.805748

**Published:** 2022-03-24

**Authors:** Maria Teresa Johnson, Julie M. Fratantoni, Kathleen Tate, Antonia Solari Moran

**Affiliations:** Center for BrainHealth, School of Behavior and Brain Sciences, University of Texas at Dallas, Dallas, TX, United States

**Keywords:** kindness, preschool, parenting, training, online, resilience, pro-social

## Abstract

A growing body of research has suggested that high levels of family functioning—often measured as positive parent–child communication and low levels of parental stress—are associated with stronger cognitive development, higher levels of school engagement, and more successful peer relations as youth age. The COVID-19 pandemic has brought tremendous disruption to various aspects of daily life, especially for parents of young children, ages 3–5, who face isolation, disconnection, and unprecedented changes to how they engage and socialize. Fortunately, both youth and parent brains are plastic and receptive to change. Resilience research shows that factors such as engaging in acts of kindness, developing trusting relationships, and responding compassionately to the feelings of others can help lay new neural pathways and improve quality of life. Yet, little research has investigated the effects of brain healthy parental practices of kindness with pre-school aged children. The current study examines whether an interactive, parent–child kindness curriculum can serve as a potentiator for brain health as measured by resilience and child empathy levels. During a peak of the pandemic, mother participants between the ages of 26–46 (*n* = 38, completion rate 75%) completed questionnaires on parental resilience levels and parent-reported child empathic pro-social behaviors before and after engaging in a 4 weeks online, self-paced, kindness curriculum. Half of the group received additional brain health education explaining the principles of neuroplasticity, empathy, perspective taking, and resiliency. Mothers in both groups showed increased resilience ( *p* < 0.001) and reported higher levels of empathic behavior in their child ( *p* < 0.001) after completing the curriculum. There was no significant difference between groups. Comparison of mean resilience levels during COVID-19 to pre-pandemic general means indicated that mothers are reporting significantly lower levels of resilience as well as decreased empathetic behaviors in their children. These results support the notion that kindness is a powerful brain health booster that can increase resilience and empathy. This research study was timely and relevant for parents in light of the myriad of stresses brought about by the ongoing COVID-19 pandemic. There are broader public health implications for equipping individuals with tools to take a proactive and preventative approach to their brain health.

## Background/Introduction

The COVID-19 pandemic permeates family functioning and wellbeing, potentially leading to a significant negative impact on parents and their young children. Parents are facing imminent threats to their relationships, social support networks, and educational access for their children, leading to overwhelming feelings of worry, stress, and anxiety (Prime et al., 2020). Specifically, the parent–child relationship is of utmost concern. Recent studies suggest that parents experiencing pandemic-related fears may have difficulty managing negative emotions, which in turn, affects daily life, family discord, and ultimately, the parent–child relationship (Daks et al., 2020; Di Crosta et al., 2020; Prikhidko et al., 2020; Saladino et al., 2020). As such, parenting young children can be challenging in and of itself, and now parents must combat additional stressors (i.e., financial, childcare, and health) due to the pandemic.

Studies indicate that, during crises, resilience (i.e., an individual’s ability to positively adapt in the face of adversity; Herrman et al., 2011) reported by women with children is considerably lower than during non-crises times and that stress levels are reported to be exacerbated (Avery et al., 2021; Taylor et al., 2021). A recent study investigating the relationship of social stressors and parent–child engagement during the COVID-19 pandemic, found that mothers and fathers who reported more social stressors were less engaged with their children and their children exhibited more behavior problems compared to before the pandemic (He et al., 2021). Fortunately, it has been shown that when parents maintain positive, responsive styles of caregiving, they can prevent and even reverse toxic levels of stress in the home caused by adversity (Blair and Raver, 2016). Stern and Cassidy (2018) found that the parent–child bond can be strengthened through an acknowledgment of empathic pro-social behaviors such as care and concern for others, which involves the capacity to comprehend the minds of others, to feel emotions outside one’s own, and to respond with kindness to others’ suffering.

Furthermore, resilience research indicates that factors such as engaging in acts of kindness, developing trusting relationships, and responding compassionately to the feelings of others can help lay new neural pathways and improve quality of life (Haslip et al., 2019). Kindness, defined as actions intended to benefit others (Curry et al., 2018) and considered as a pro-social relational construct, supports an intra and interpersonal focus on how one treats others, takes care of oneself, and interacts with the world around them. As such, parent-driven kindness interventions may prove fruitful in promoting resilience, as parents have the influence and opportunity to become the first teachers and models for acts of kindness with their children.

The global pandemic has put a spotlight on brain health and the great need for resources, education, and training. Brain health is defined as a state of performing at your personal best and thriving in your life context—not simply the absence of disease. The term brain health as described by Chapman et al. (2021) holistically encompasses the brain’s functions which includes aspects of cognition (ex: problem solving, innovation, processing speed, and memory), daily life (ex: responsibilities, sleep, nutrition, and exercise), wellbeing (ex: resilience, quality of life, and mood), social interaction (ex: empathy, kindness, and social support), and neural components (ex: brain blood flow and connectivity). In contrast, mental health is a term that more narrowly focuses on psychological and emotional wellbeing. Recent work has highlighted how the different components of brain health influence each other and by strengthening skills in one area, may also compensate for areas of weakness. A case study showed that after completing an online cognitive intervention, some of the outcomes were that the participant felt more satisfied with her social networks and saw improvement in measures of wellbeing, which included increased resilience and decreased stress (Chapman et al., 2021). Following this line of thinking, the current study seeks to understand how a kindness intervention may improve resilience.

With social distancing and stay-at-home mandates in effect, digital tools that are easily accessible and cost effective offer a solution to help families navigate the stresses of the COVID-19 pandemic. Studies have demonstrated the value of digital interventions in allowing various populations, including families, to access evidence-based guidance on demand and through a modality (web-based) that they are already comfortable using to seek mental, behavioral, and brain health guidance (Lund et al., 2018; Caulfield and George, 2020; O’Dell et al., 2021). Supplementary to intervention, other tools such as self-paced, at-home brain science education, could offer additional insight for parents seeking to better understand their own brain health. Yet, currently, there is limited data on the effects of brain science education on the resilience levels of parents with young children amid a pandemic. This study seeks to understand if an online kindness training may increase resilience in parents with preschool-aged children, promote empathic pro-social behavior in their children, and parents find the kindness activities relevant.

## Aims and Hypothesis

Given the timely need for at-home parenting programs that support the social, emotional, and relational emergence of developing young minds, collaborators from the University of Texas at Dallas Center for BrainHealth, alongside the Children’s Kindness Network, based in TN, had a specific interest in the impact of Kind Minds with Moozie, an online kindness training for parents of preschoolers. The aim of this study is to understand if practicing the pro-social skills of kindness may (1) affect resilience in parents and (2) affect empathic pro-social behavior in preschool-aged children.

The hypothesis for this study was that (1) parents who engage with Kind Minds with Moozie will increase resilience and observe increased empathic pro-social behaviors in their child, (2) additional brain science education for the parents would contribute to greater gains in resilience, and (3) parents would find kindness activities relevant during interactions with their preschool-aged children.

## Materials and Methods

### Procedure and Study Design

Participants were randomized into either the Kindness Only condition, or the Kindness with Brain Science condition *via* a simple random sample process. All individuals provided written informed consent to participate, and all procedures were approved by and carried out in accordance with the University of Texas at Dallas Institutional Review Boards, number 21-104. The study was conducted entirely online from April to July 2021. Recruitment was open to both mothers and fathers; however, most participants who enrolled in the study were mothers. One father enrolled in the study but did not complete the online modules and was considered loss to follow-up and was not included in the analysis.

### Participants

Participants were recruited for the study through professional networks and social media posts, primarily in online groups for parents. Parents with children (three to 5 years of age) were screened to determine if they qualified for the study. Participants who met all inclusion and failed to meet exclusion criteria were enrolled in the study. Inclusion criteria consisted of: the parent being 18 years of age or older, having access to the Internet (including access to a computer/smartphone/tablet), identifying as the primary caregiver/parent within the target child age range, and being a proficient English speaker. If the parent agreed, they were provided with an electronic consent form explaining the procedures for the study and provided written consent. Thirty-eight mothers with children between the ages of 3 years, 0 month, and 5 years, 11 months (*M* = 3.97 years; male = 61%, female = 39%) participated in the study. The study included mothers between the ages of 26 and 46 (*M* = 36.35 years) who were relatively highly educated (25% up to Bachelor’s, 55% Master’s, and 15% Doctorate). See Table 1 for a breakdown of ethnicity and gender for the parent participants and their children.

**Table 1:** Demographic data.

Parent Ethnicity	Frequency	Percentage
White	28	73.7
Hispanic/Latino	3	7.9
Asian/Pacific Islander	4	10.5
Other	2	5.3
Not reported	1	2.6
**Parent gender**		
Female	37	97.4
Male	0	0
Not reported	1	2.6
**Child ethnicity**		
White	27	71.0
Hispanic/Latino	3	7.9
Asian/Pacific Islander	2	5.3
**Other**	5	13.2
Not reported	1	2.6
Child gender		
Female	14	36.8
Male	22	57.9
Prefer not to say	1	2.6
Not reported	1	2.6

*Parent and child ethnicity and gender (*N* = 38)*.

## Moozie Teaches Kindness Curriculum

The *Moozie Teaches Kindness* curriculum for preschool-aged children, developed by the Children’s Kindness Network, includes do-at-home kindness activities that utilize music, art, and creativity to move methodically from the center of the child’s circle, him/herself, to the ever-widening rings of awareness of others, animals, the environment, and nature (Children’s Kindness Network, 2013). Moozie, an ambassador of kindness, is presented as a lovable, gentle, digital cow to whom children can easily relate and from whom they learn valuable, lifelong lessons. The instructional design of the *Moozie Teaches Kindness* curriculum was developed to meet National Association for the Education of Young Children (NAEYC) standards for Social–Emotional and Cognitive Development with the target age group being 3 to 7 (NAEYC, 2019).

Researchers selected and adapted the *Moozie Teaches Kindness* curriculum for this study based on its applicability to parents of preschool-aged children and focus on pro-social behaviors using the four kindness pillars that are paramount to brain health: Kindness to Others, Kindness to Self, Kindness to Animals, and Kindness to Earth. Each kindness pillar teaches parents how they can contribute to the development of empathic pro-social behavior of their child through parent-led activities which promote recognizing and naming feelings of self and others, sharing, taking turns, helping others, saying kind words, interacting with pets and/or outdoor animals, and being kind to nature in positive (recycling and conserving) and negative ways (littering and wasting).

### Kind Minds With Moozie Protocol

Kind Minds with Moozie was a randomized, pilot intervention trial designed to examine benefits of an online kindness training protocol for parents and their preschoolers. Accessed *via* parent’s electronic device (laptop, phone, tablet, and desktop computer) parent participants completed five online kindness modules, each designed to take less than 10 min to complete. Parents were asked to click through a series of written and pictorial step-by-step kindness activities to be later implemented when interacting with their children (Tables 2, 3). Participants in the study were randomly assigned to one of two conditions and subsequently completed pre-test measures, online modules with kindness content and post-module surveys, and then post-test measures within 1 week of completion of the last online kindness module.

**Table 2:** Kind minds modules based on the four pillars of kindness.

Kind minds online modules	Kindness only content	Kindness with brain science content
Overview	Introduction to Moozie	Introduction to Moozie
Kindness to others	Cultivating Kindness	Cultivating Kindness and Empathy and Mirror Neurons
Kindness to self	Practicing Kindness	Practicing Kindness and Self-Compassion and Parasympathetic Nervous System
Kindness to animals	Modeling Kindness	Modeling Kindness and Resilience and The Frontal Lobe
Kindness to nature	Spreading Kindness	Spreading Kindness and Neuroplasticity and Flexibility

*The kind minds online modules provided education on the four pillars of kindness with additional brain science for one condition*.

**Table 3:** Online kindness activities.

Kind minds online modules	Prompt from Moozie	Kindness activity 1	Kindness activity 2	Kindness activity 3
Kindness to others	“What can you do today to be kind to our family and neighbors?”	Remind your child that being kind is important to our own well-being and to that of others.	Play “Catch a Smile” with your child. How many times can you catch each other smiling? Write down this number each day and throughout your week. Then, see if you can make it grow!	Play a game of charades with your child. Take turns acting out the different ways you showed kindness this week. Try to guess each other’s kind acts.
Kindness to self	“Good morning!” to yourself with kindness and a smile.	Encourage your child to start off the day best by saying this to themselves in front of a mirror. Remind them that taking care of their hair, teeth, and body, is being kind to themselves, too!	Play “Moozie Munchies” with your child. How many times can you make healthy food choices this week? Each day, draw a picture of all the fruits and veggies you ate, then watch your picture garden grow!	Take a walk with your child. Use all of your senses to talk about what you see, hear, feel, smell, and touch. Remind your child that physical activity is a very important part of being kind to yourself.
Kindness to animals	“I am sleepy. Moo. Please wish me and all my animal friends a sweet and dream-filled rest.”	Share with your child that animals are kind to us, too, and we can be kind to them. Point out that our day always starts with birds singing in the morning. What is your favorite song?	Guide your child in placing a bowl(s) of water outside for our animal friends (on a fire escape, in the park, or in the yard). Try to remember to refill the bowl each day, then see what animal friends come to visit!	Parents describe how when we love a family member, we often give them hugs. We like to hug, pet, and play with our pets, too! Choose a stuffed animal (or your pet!) and practice kindness by giving and receiving lots of love.
Kindness to nature	“Let us walk through a park or a backyard and find gifts from nature like a flower, a cloud, a blade of grass, or a unique rock.”	Play “Picture Perfect” with your child. Grab a camera or a sketchbook and look out the window sometime during the morning. Draw or take a picture of the world around you. Have your child tell a story about their picture.	Remind your child that nature is a gift. Invite your child to go on a “Trash or Treasure Hunt” with you and find all the special gifts outdoors that can easily be overlooked. Shift your perspective to see the magic happening all around you. Be kind to the earth by removing any real trash during your explorations.	Have your child interact with the digital garden. Be sure to remind your child that plants and flowers have special powers – they help take care of you, each other, animals, and our planet! Moo!

*The parent-led activities to be completed with their preschoolers*.

### Kindness Only Condition

The first kindness only condition (*n* = 17) included an overview module introducing Moozie as the ambassador of kindness and setting a learn, do, and reflect pedagogy. This pedagogy introduced parents to the pillars of kindness (learn), described steps to and importance of including kindness in daily parenting activities (do), and prompted parents to consider the likelihood of integrating a kindness focus into their parenting style (reflect). Each of the modules provided graphics, clickables, and simple activities to engage parents. On average, it took parents 29.25 min to complete all five modules over a period of 4 weeks.

### Kindness With Brain Science Condition

The second kindness with brain science condition (*n* = 21) included the same overview and online kindness modules as the first condition, as well as a brief brain science component during the learning stage. Each brain science learning component consisted of 2–3 additional paragraphs of reading material describing empathy, resilience, neuroplasticity, and flexibility. This additional brain science was provided to explain the importance, “the why” of each concept to overall parental brain health. Participants in this condition were not informed that they would be receiving this additional content. On average, it took parents 33.14 min to complete all five modules over a period of 4 weeks.

### Measures

Resilience was measured using the self-report 25-item Connor-Davidson Resilience Scale (CD-RISC; Connor and Davidson, 2003). The scale has been developed and tested as a measure of the degree of resilience and has promise as a method to screen people for high, intermediate, or low resilience. The total score can range from 0 to 100 and the higher the score obtained, the greater the participants resilience. Each parent rated their own stress coping ability on a 5-point scale (0–4), with higher scores reflecting greater resilience in areas such as an individual’s ability to adapt when changes occur, staying focused and thinking clearly when under pressure, and bouncing back after injury, illness, or hardship. The CD-RISC measure of resilience normative data indicates that the US general population median score is 82, with the first quartile (Q1: 0–73) describing the score range for the lowest group (lowest 25% of the population), i.e., the least resilient, the second (Q2: 74–82) and third (Q3: 83–90) the intermediate scores, and the fourth (Q4: 91–100) describing the highest or most resilient, i.e., above 75% of the population. This measure is found to have a very good internal consistency as measured by Cronbach’s α (*α* = 0.93).

Empathic pro-social behavior was measured using a National Institute of Health (NIH) Toolbox Empathic Behaviors Survey CAT Ages 3–13 v2.0 (EBS), a parent-report measure for children ages 3 through 12 that assesses parent perceptions of children’s pro-social behaviors using a 10-item fixed length form. The EBS is a specific test within the NIH Toolbox—Emotion—Social Relationships—Positive Social Development (Salsman et al., 2013). This parental proxy scale was developed to assess early behavioral indicators of positive social development (i.e., empathic pro-social behaviors). Each item administered has a 5-point scale with options ranging from never to always. An example of a parent’s perception of the child’s empathic pro-social behavior would be “In the past month, please decide: How often your child offers to help other children who are having difficulty.” Higher scores are indicative of more parent reported child pro-social behaviors, with a normative mean T-score of 50. This measure is found to have a very good internal consistency as measured by Cronbach’s α (*α* = 0.90).

Relevancy of the program was measured using a 5-point Likert scale (1 = strongly disagree and 5 = strongly agree) that parents completed after each of the online kindness modules. These five, three-question surveys asked parents to reflect and rate their experience in terms of content comprehension, relevance to parenting style, and likelihood of implementing the kindness practice into daily life (Table 4). This relevancy survey was developed by the Kind Minds researchers to examine the saliency of this training for parents. Examples of the relevance questions include “I understand how being kind to others plays a role in having a kind mind” (comprehension), “I find the concept of compassion relevant to my parenting style” (relevance), and “I will practice modeling and expressing empathy with my child” (likelihood).

**Table 4:** Relevancy survey questions.

Relevancy survey questions		
Comprehension of concept	Relevancy of concept	Practice the concept with my child
Kindness only condition		
I understand that this study aims to equip and empower me to be a kind, parenting mind.	I find the concept of kindness relevant to my parenting style.	I will practice Moozie moments and kindness with my child.
I understand how being kind to each other plays a role in having a kind, parenting mind.	I find the concept of modeling kindness relevant to my parenting style.	I will practice showing kindness to my child.
I understand how being kind to myself plays a role in having a kind, parenting mind.	I find the concept of practicing self-care relevant to my parenting style.	I will practice self-care with my child.
I understand how being kind to earth plays a role in having a kind, parenting mind.	I find the concept of being kind to earth relevant to my parenting style.	I will practice being kind in nature with my child.
I understand how being kind to animals plays a role in having a kind, parenting mind.	I find the concept of practicing kindness to animals relevant to my parenting style.	I will practice kindness towards animals with my child.
**Kindness with Brain Science Condition**		
I understand that this study aims to equip and empower me to be a kind, parenting mind.	I find the concept of kindness relevant to my parenting style.	I will practice Moozie moments and kindness with my child.
I understand how empathy plays a role in having a kind, parenting mind.	I find the concept of empathy relevant to my parenting style.	I will practice modeling and expressing empathy with my child.
I understand how compassion plays a role in having a kind, parenting mind.	I find the concept of compassion relevant to my parenting style.	I will practice self-compassion and calming exercises with my child.
I understand how neuroplasticity plays a role in having a kind, parenting mind.	I find the concept of flexibility relevant to my parenting style.	I will practice being flexible with my child.
I understand how resilience plays a role in having a kind, parenting mind.	I find the concept of resilience relevant to my parenting style.	I will practice resiliency with my child.

*The participants were asked to rate their comprehension, the relevancy, and the likelihood of practicing the concept at the end of each online kindness module*.

## Results

To test the hypotheses that parents who engage with Kind Minds with Moozie would increase resilience and observe increased empathic pro-social behaviors in their child, a paired sample t-test was conducted. Secondly, a two-sample t-test was conducted to determine the effects of additional brain science education on resilience levels. Lastly, to test the hypothesis that parents would find kindness activities relevant during interactions with their preschool-aged children, post-training participant ratings were collected and averaged. All statistics were done in SPSS (IBM Corp., 2019).

Toward completion of the study activities, researchers recommended parents complete one online kindness module per week and activated a 5-day time lapse between the completion of one module and access to the next, thereby allowing sufficient time for practice of the kindness activities with their children and subsequent completion of the relevancy surveys. On average, participants took 34.7 days to complete the study from pre-test date to post-test date.

### Resilience

At baseline (T1), both conditions rated low levels of resilience, with both groups falling within the first quartile (Q1: 0–73). Post-training (T2), mothers in both conditions increased their mean scores to an intermediate level of resilience, falling within the second quartile (Q2: 74–82); the kindness with brain science condition reported slightly higher levels of resilience than the kindness only condition (Table 5). A paired sample t-test showed a whole group significant increase in resilience (*p* < 0.001) after completing the online kindness modules (Table 6).

**Table 5:** Connor-Davidson resilience scale and NIH toolbox empathic behaviors survey means.

	M	SE	n
CD-Risc Kindness Only T1	69.59	11.34	17
CD-Risc Kindness Only T2	75.24	11.52	17
CD-Risc Kindness with Brain Science T1	69.67	8.00	21
CD-Risc Kindness with Brain Science T2	76.57	10.86	21
EBS Kindness Only T1	43.44	8.56	17
EBS Kindness Only T2	49.82	8.57	17
EBS Kindness with Brain Science T1	42.45	10.13	21
EBS Kindness with Brain Science T2	47.06	12.88	21

*A table of descriptive statistics displays the averages of participant scores in both conditions, kindness only and kindness with brain science, at T1 and T2*.

**Table 6:** Paired sample *t*-test for Connor-Davidson resilience scale and NIH toolbox empathic behaviors survey.

	M	SE	t	df	p
CD-Risc T1-T2 Whole Group	−6.34	8.89	−4.39	37	0.000**
CD-Risc T1-T2 Kindness Only	−5.64	2.11	−2.66	16	0.017*
CD-Risc T1-T2 Kindness with Brain Science	−6.90	2.00	−3.44	20	0.003*
EB T1-T2 Whole Group	−5.40	5.16	0.83	37	0.000**
EB T1-T2 Kindness Only	−6.38	1.23	−5.18	16	0.000**
EB T1-T2 Kindness with Brain Science	−4.60	1.14	4.03	20	0.001**

*Paired sample t-tests revealed significant differences in reported means from T1 to T2 in both the kindness only and the kindness with brain science conditions*.

* *p < 0.05*.

** *p < 0.001*.

### Empathic Pro-Social Behavior

Prior to the training (T1), mothers reported child empathic pro-social behaviors levels below expected norms (T < 50). Upon post-test (T2), mothers in both groups rated their perception of their child’s empathic pro-social behaviors as significantly increased, with the kindness only condition outperforming the kindness with brain science condition (Table 5). A paired *t*-test revealed mothers in both groups reported observing higher levels of empathic pro-social behavior in their child (*p* < 0.001) after completing the online kindness modules (Table 6).

### Brain Science

A two-sample t-test found no significant differences in CD-RISC between the kindness only and kindness with brain science conditions (Table 7).

**Table 7:** Two-sample t-test comparing kindness with brain science to the kindness only condition.

	M(T1-T2)	SE(T1-T2)	t	df	p
CD-Risc Kindness with Brain Science-Kindness only	1.25	2.93	0.429	36	0.671

*The two-sample *t*-test demonstrated that there was no significant difference in the change reported from T1 to T2 in the kindness only and kindness with brain science conditions on the CD-Risc*.

### Relevancy

The mean relevancy scores in both groups revealed that mothers reported overall high relevancy after completing each of the online kindness modules (Table 8). Responses ranged from 4.69 to 4.91 out of a 5-point Likert scale (1 = strongly disagree and 5 = strongly agree).

**Table 8:** Relevancy survey questions.

Relevancy survey means			
Online kindness modules	Understand the concept	Find the concept relevant	Practice the concept with my child
Kindness overview (*n* = 33)	4.91	4.85	4.73
Kindness to others and Empathy (*n* = 32)	4.84	4.88	4.81
Kindness to self and self-compassion (*n* = 33)	4.88	4.85	4.73
Kindness to animals and resilience (*n* = 35)	4.75	4.78	4.74
Kindness to nature and neuroplasticity (*n* = 32)	4.78	4.69	4.88

*The participants were asked to rate their comprehension, the relevancy, and the likelihood of practicing the concept at the end of each online kindness module*.

## Discussion

The Kind Minds with Moozie research study sought to understand if an online kindness curriculum could be a potentiator for resilience and empathic pro-social behavior during times of stress brought about by the COVID-19 pandemic. One aim of this study was to integrate easy-to-follow brain science education with kindness activities delivered digitally. We hypothesized that parents who engaged with the online kindness activities with their preschool-aged children would boost parental levels of resilience and parent reported child empathic pro-social behavior levels. Results showed a whole group increase in resilience levels of mothers and mother-reported empathic pro-social behaviors in their children. This study supports the notion that practicing kindness can be a useful tool to help mothers become more resilient. The ability to overcome difficulties and cope with stress is critical, especially during a global pandemic. As such, changes in resilience, a personality trait aimed at complying with environmental changes and stress, may be a beneficial factor to consider (Block and Block, 1980). There is a need for additional research and salient early interventions for parents, including both mothers and fathers, as resilience can be a potentiator for improved mental, physical, and brain health.

Given that the baseline resilience levels of mothers in this study fell within the bottom 25% of the population (*m* = 69), there was opportunity for growth and intervention. One possible explanation is that the pandemic contributed to feelings of worry and fear, which may then affect mothers’ resilience levels. Similar to the findings of our study, Mariani Wigley et al. (2020) used the same measure of resilience and investigated the support role of parents during the COVID-19 emergency. Results showed that parents were also found to have a low parental resilience score (*m* = 63.78) when their children were on average 8 years of age. Compared to the mothers of preschoolers in this study, who reported higher resilience levels before and after the kindness training (*m* = 69, *m* = 75.9), our results suggest that maternal resilience levels may fluctuate not only due to environmental stressors, but also depending on child age. Therefore, implementing a kindness training during the earlier years of childhood may serve as a buffer against declining parental ability to adapt and bounce back in the face of stressful situations.

Prior to receiving this online kindness training, mothers in both the kindness only and kindness with brain science conditions reported child empathic pro-social behaviors at levels lower than expected norms (T < 50). Upon completion of the online kindness modules, a significant increase in whole group child empathic pro-social behaviors was reported (*m* = 48.30), although the scores increased, they were still slightly below the norm. One potential factor for consideration is that these low scores may be due to the isolating nature of the COVID-19 pandemic as children might be restricted from engaging in social–emotional learning activities outside of the home or have limited social engagement with peers in order for natural development of pro-social behavior through activities involving same-aged play, peer modeling, and social communication.

Regarding differences between the kindness only and the kindness with brain science conditions, the authors hypothesized that both groups would demonstrate gains in resilience and parent reported empathic pro-social behavior and that the participants in the kindness with brain science condition would show greater increases in parental resilience. Analysis revealed both groups did increase in resilience and parent reported empathic pro-social behavior in their children; however, there was no significant difference between groups. One potential reason for this finding may be that the measures were not well suited to capture the impact of the brain education provided. We did not include application questions for the brain science information provided. Research has shown that synthesis (gist reasoning) is an important process to abstract meaning from complex information and that gist reasoning can predict performance in daily function (Vas et al., 2015). Providing information alone may not have been enough to create measurable changes in resilience. Future studies should investigate the possibility of making the brain science educational aspect more thorough, with specific and direct applications. Mothers reported high relevancy upon completion of the online kindness modules. Study participants reported they found Kind Minds with Moozie to be comprehensible, relevant, and practical. Additionally, on average, parents in the kindness only condition spent 29.25 min and parents in the kindness with brain science condition took slightly longer at 33.14 min to complete the entire course over the course of 4 weeks. Given that the additional brain science education should have only resulted in a brief increase in the amount of time taken to complete each module, this small difference is expected. Nonetheless, with the time-consuming demands placed on parents during the pandemic, it is promising that this brief, online kindness training can be completed in less than 1 h. Furthermore, the results of this study suggest that mothers value practicing and instilling pro-social skills such as being kind to others, yourself, animals, and nature in their children and that kindness activities, which foster parent–child interaction, are well received.

### Limitations and Future Directions

This research study has several strengths and limitations related to the study design. Due to the limitations of the study, the results must be interpreted with caution. The two conditions of the study allowed for examination of the added benefits of brain science to online kindness activities; however, the study could benefit from a third condition including parents who would not receive the brain science. While a control group which would receive materials after the post-intervention measurement of resilience and empathic pro-social behaviors could have provided additional insights into the effectiveness of the online kindness training, the research team prioritized delivering the training in a timely manner due to the pandemic. This online kindness training was relevant for mothers considering the myriad of stresses and demands brought about by the ongoing COVID-19 pandemic. The digital design of the study was an efficient method for researchers to provide study activities to participants during a period of physical and social distancing, although participant feedback on the accessibility and ease of use of the technology was not collected. Additionally, the participant feedback surveys gathered insight regarding the comprehension, relevancy, and practicality of the kindness activities in daily life; however, the feedback did not address parent engagement levels or frequency of practicing the kindness activities with their children. These aspects could be assessed in future studies to gain additional information which would be useful for the implementation of the program and the evaluation of its feasibility. In regard to the participants, it was a homogeneous group, as more female participants enrolled in the study, and many came from similar educational and socioeconomic levels; therefore, the data collected were limited in representation to mothers of preschool-aged children. The study design could be strengthened by adding a follow-up time point to assess maintenance effects of gains in parent resilience and child pro-social behavior. Further exploration of how a more structured cognitive training combined with daily habits may affect greater change in parent resilience levels may be of interest in a larger-scale investigation. Continued effort to expand and enroll a third control group would lend itself to a more robust analysis of the impact and effects of brain science education on resilience, empathy, and cognition. Future recruitment processes should include a more focused diversification so that multiple demographics and both maternal and paternal figures are represented. Overall, study findings serve as a model for leveraging a neuroscience-based online kindness curriculum to empower parents with strategies to combat stress exacerbated by these unique times. There are broader public health implications for equipping individuals with tools to take a proactive and preventative approach to brain health, thereby influencing the social, academic, and neural development of the family unit (Feldman, 2015). The chronic and cumulative effects of stress on the brain can contribute to adverse childhood experiences and have been linked to parental resilience as a mediator. Borja et al. (2019) suggest that the resilience of some parents can prevent the heightened exposure of their children to adversities.

Continued studies should further investigate specific methods and protocols utilizing kindness and resilience building activities that promote parent–child interaction and relational development as a foundation to creating happier and more brain healthy families.

## Conclusion

Identifying effective ways to reduce stress and increase resilience has become a mandate for people from a myriad of life, age, professional, and socioeconomic backgrounds, and especially among parents and their young children. Kindness is a familiar construct that goes beyond educational, psycho-social, and cultural boundaries; however, many current practices do not involve a curriculum devised specifically for the implementation by parents of preschool-aged children. The developing mind is instrumental in instilling strong, neural pathways that promote resilience and empathic pro-social behavior. Kind Minds with Moozie resulted in a valuable tool to provide structured support and didactic instruction to assist parents in supporting and promoting child empathic pro-social behavior and proved to be useful in support interventions for families exposed to adverse events as well as public health crises. Specifically, Kind Minds with Moozie could be used to plan intervention for caregivers (e.g., teachers and parents) aimed at improving resources to cope with life stressors. Thus, the present results highlight the significance of designing digital therapeutic tools and kindness training designed to improve both parental and child wellbeing.

## Data Availability Statement

The raw data supporting the conclusions of this article will be made available by the authors, without undue reservation.

## Ethics Statement

The studies involving human participants were reviewed and approved by University of Texas at Dallas. The patients/participants provided their written informed consent to participate in this study.

## Author Contributions

MJ co-designed the study, conducted recruitment, created the online modules, enrolled and managed participants, scored and interpreted data, and wrote the manuscript. JF co-designed the study, assisted with recruitment, and manuscript preparation and edits. KT assisted with data interpretation and manuscript preparation and edits. AM assisted with participant screening and recruitment, and manuscript edits. All authors contributed to the article and approved the submitted version.

## Funding

The Heppner Brain Research in Children’s Kindness supported by HERO and the Beneficient Trust Company funded the Kind Minds study. We are deeply grateful to HERO and the Beneficient Trust Company’s financial support.

## Conflict of Interest

The authors declare that the research was conducted in the absence of any commercial or financial relationships that could be construed as a potential conflict of interest.

## Publisher’s Note

All claims expressed in this article are solely those of the authors and do not necessarily represent those of their affiliated organizations, or those of the publisher, the editors and the reviewers. Any product that may be evaluated in this article, or claim that may be made by its manufacturer, is not guaranteed or endorsed by the publisher.
